# Supplementary material for: The Evolution and Characterization of the RNA Interference Pathways in Lophotrochozoa
Source: Genome Biol Evol. 2024 May 7;16(5):evae098. doi: 10.1093/gbe/evae098 (PMC11114477; doi:10.1093/gbe/evae098)
Supplement: evae098_Supplementary_Data [file evae098_supplementary_data.zip › Supplementary_materials.zip!/selected_miRNAs/NoGe_selected_miRNAs/mirdeep_pdf_files/NMRB01000380.1_Notospermus_geniculatus_strain_Ushimado_scaffold380,_whole_genome_shotgun_sequence_11928.pdf]

Provisional ID : NMRB01000380.1_Notospermus_geniculatus_strain_Ushimado_scaffold380,_whole_genome_shotgun_sequence_11928
Score total : 200834.2
Score for star read(s) : 3.9
Score for read counts : 200825.3
Score for mfe : 2
Score for randfold : 0
Score for cons. seed : 3
Total read count : 393922
Mature read count : 393541
Loop read count : 0
Star read count : 381

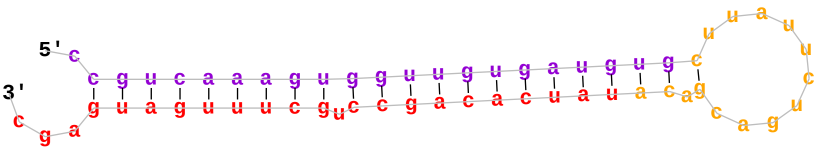

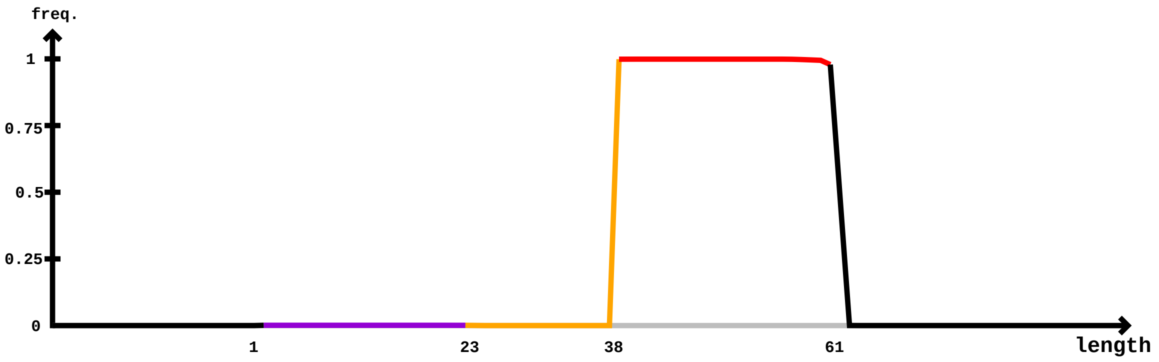

Star Mature

```
5'- uguuugagucuucgucuaguccgucaaagugguugugaugugcuuauucugacgacauaucacagccugcuuugaugagcuggauguuagaguggacuacagucaaaugc  -3'   obs
    uguuugagucuucgucuaguccgucaaagugguugugaugugcuuauucugacgacauaucacagccugcuuugaugagcuggauguuagaguggacuacagucaaaugc         exp
    (((((.(.(((.((((((((.((((((((((((((((((((((..........).)))))))))))).))))))))).))))))))..))).).))..))).........   reads   mm   sample
    ....................ccgucaaagugguugugaug......................................................................   17      0    Fm3
    ....................ccgucaaagugguugugCugug....................................................................   1       1    Fm3
    ....................ccgucaaagugguuguUaugug....................................................................   1       1    Fm3
    ....................ccgCcaaagugguugugaugug....................................................................   1       1    Fm3
    ....................ccgucaaagugguugugaugug....................................................................   158     0    Fm3
    ....................ccgucaaaguAguugugaugug....................................................................   1       1    Fm3
    ....................ccgucaaagugguugugaugugU...................................................................   7       1    Fm3
    .........................................................uaucacagccugcuuuUa...................................   1       1    Fm3
    .........................................................uaucacagccugcuuugU...................................   13      1    Fm3
    .........................................................uaucacagccugcuuuAa...................................   1       1    Fm3
    .........................................................uaucacagccugcuuuCa...................................   1       1    Fm3
    .........................................................uaucacagccugcuuuga...................................   79      0    Fm3
    .........................................................uaucacagccugcuuugCu..................................   1       1    Fm3
    .........................................................uaucacagccugcuuuAau..................................   2       1    Fm3
    .........................................................uaucacagccugcuuugau..................................   201     0    Fm3
    .........................................................uaucacagccugcuuugUu..................................   16      1    Fm3
    .........................................................uaucacagcAugcuuugau..................................   1       1    Fm3
    .........................................................uaucacagccugcuuugaA..................................   3       1    Fm3
    .........................................................uaucacagccugcuuugGu..................................   1       1    Fm3
    .........................................................uaucacagUcugcuuugau..................................   2       1    Fm3
    .........................................................uaucacagUcugcuuugaug.................................   5       1    Fm3
    .........................................................uaucacagccugcuuugGug.................................   1       1    Fm3
    .........................................................uaucacagccugcuuugauC.................................   1       1    Fm3
    .........................................................uaucacagccugcuuugCug.................................   1       1    Fm3
    .........................................................uaucacagccugcuuugauA.................................   7       1    Fm3
    .........................................................uaucacagccGgcuuugaug.................................   1       1    Fm3
    .........................................................uaucacagccugcuuugauU.................................   5       1    Fm3
    .........................................................uaucacagccugcuuugaAg.................................   4       1    Fm3
    .........................................................uaucacagccugcuuugaug.................................   209     0    Fm3
    .........................................................uaucacagccugcuuuUaug.................................   2       1    Fm3
    .........................................................uaucacagccugcuuugaCg.................................   1       1    Fm3
    .........................................................uaucacagUcugcuuugauga................................   4       1    Fm3
    .........................................................uaucacagccugcuuugaCga................................   2       1    Fm3
```

```
                     Star                                 Mature
uguuugagucuucgucuaguccgucaaagugguugugaugugcuuauucugacgacauaucacagccugcuuugaugagcuggauguuagaguggacuacagucaaaugc
.........................................................uaucacagccugcuuugauga................................  169    0  Fm3
.........................................................uaucacagccugcCuugauga................................  4      1  Fm3
.........................................................uaucacagccugcuuugaugU................................  14     1  Fm3
.........................................................uaucacagccugcuuuUauga................................  1      1  Fm3
.........................................................uaucacagccugcuuugaugag...............................  1981   0  Fm3
.........................................................uaucacagccugcuuugaugaU...............................  6      1  Fm3
.........................................................uaucacagccugcuuugauUag...............................  22     1  Fm3
.........................................................uaucacagccugUuuugaugag...............................  6      1  Fm3
.........................................................uaucacagccuAcuuugaugag...............................  1      1  Fm3
.........................................................uaucacagccugcuuugGugag...............................  5      1  Fm3
.........................................................uaucacagccugcuuugaugUg...............................  3      1  Fm3
.........................................................uauAacagccugcuuugaugag...............................  1      1  Fm3
.........................................................uaucacGgccugcuuugaugag...............................  3      1  Fm3
.........................................................uaucacagccugcuuuCaugag...............................  2      1  Fm3
.........................................................uaucacagccCgcuuugaugag...............................  2      1  Fm3
.........................................................uaCcacagccugcuuugaugag...............................  1      1  Fm3
.........................................................uaucacagccugcuuugUugag...............................  2      1  Fm3
.........................................................uaucaUagccugcuuugaugag...............................  1      1  Fm3
.........................................................uaucacagccugcuuGgaugag...............................  5      1  Fm3
.........................................................uaucacagccugcAuugaugag...............................  3      1  Fm3
.........................................................uaucacagccGgcuuugaugag...............................  3      1  Fm3
.........................................................uGucacagccugcuuugaugag...............................  2      1  Fm3
.........................................................uauGacagccugcuuugaugag...............................  1      1  Fm3
.........................................................uaucacagccugcuuugCugag...............................  10     1  Fm3
.........................................................uaucacagccugcuuuUaugag...............................  7      1  Fm3
.........................................................uaucacagccugcuCugaugag...............................  3      1  Fm3
.........................................................uaucacagccugcuuCgaugag...............................  1      1  Fm3
.........................................................uaucacaUccugcuuugaugag...............................  2      1  Fm3
.........................................................uaucacagcUugcuuugaugag...............................  1      1  Fm3
.........................................................Aaucacagccugcuuugaugagc..............................  5      1  Fm3
.........................................................uaucacagccugcuGugaugagc..............................  6      1  Fm3
.........................................................uaucacagccugcuuAgaugagc..............................  7      1  Fm3
.........................................................uaucacagccugcuCugaugagc..............................  9      1  Fm3
.........................................................uaucacNgccugcuuugaugagc..............................  5      1  Fm3
.........................................................uaGcacagccugcuuugaugagc..............................  20     1  Fm3
.........................................................uaucacagccugcuuugaAgagc..............................  2      1  Fm3
.........................................................uaucacagccugcuuugaugagc..............................  89646  0  Fm3
.........................................................uaucaNagccugcuuugaugagc..............................  15     1  Fm3
.........................................................uaucCcagccugcuuugaugagc..............................  18     1  Fm3
.........................................................uaucUcagccugcuuugaugagc..............................  8      1  Fm3
.........................................................uaucacagccugcGuugaugagc..............................  8      1  Fm3
.........................................................uaucacagUcugcuuugaugagc..............................  87     1  Fm3
.........................................................uaucacagccugGuuugaugagc..............................  1      1  Fm3
.........................................................uaucacagccugcuuugaugagA..............................  38     1  Fm3
.........................................................uaucacagccugcuuugaugaAc..............................  7      1  Fm3
.........................................................uaAcacagccugcuuugaugagc..............................  24     1  Fm3
.........................................................uaucaGagccugcuuugaugagc..............................  10     1  Fm3
.........................................................uaucacagccugcuuugaugagU..............................  176    1  Fm3
.........................................................uaNcacagccugcuuugaugagc..............................  1      1  Fm3
.........................................................uaucacagccugcuuugaugUgc..............................  21     1  Fm3
.........................................................uGucacagccugcuuugaugagc..............................  11     1  Fm3
.........................................................uUucacagccugcuuugaugagc..............................  16     1  Fm3
.........................................................uaucacagccugcuuugauUagc..............................  42     1  Fm3
.........................................................uaucacagccugcuuuAaugagc..............................  174    1  Fm3
.........................................................uaucacagccugcuAugaugagc..............................  14     1  Fm3
.........................................................uaucacagccugcuuuUaugagc..............................  129    1  Fm3
.........................................................uaucacagcUugcuuugaugagc..............................  14     1  Fm3
.........................................................uaucacagccugcuuGgaugagc..............................  5      1  Fm3
.........................................................uaucacagccugcAuugaugagc..............................  20     1  Fm3
.........................................................uaucacagccugcuuugUugagc..............................  294    1  Fm3
.........................................................uaucacagccugcuuuNaugagc..............................  1      1  Fm3
.........................................................uaucacagccugcuuugaugaUc..............................  22     1  Fm3
.........................................................uaucacagccuCcuuugaugagc..............................  4      1  Fm3
.........................................................uaucacaUccugcuuugaugagc..............................  28     1  Fm3
.........................................................uaucacagccugcCuugaugagc..............................  30     1  Fm3
.........................................................uaucacagccugcuuugaGgagc..............................  3      1  Fm3
.........................................................uaucacCgccugcuuugaugagc..............................  1      1  Fm3
.........................................................uaucacaCccugcuuugaugagc..............................  9      1  Fm3
.........................................................uaucacagGcugcuuugaugagc..............................  5      1  Fm3
.........................................................uauUacagccugcuuugaugagc..............................  16     1  Fm3
```

```
                    Star                                Mature
uguuugagucuucgucuaguccgucaaagugguugugaugugcuuauucugacgacauaucacagccugcuuugaugagcuggauguuagaguggacuacagucaaaugc
........................................................uaucGcagccugcuuugaugagc..............................  15    1  Fm3
........................................................uaucacagcAugcuuugaugagc..............................  9     1  Fm3
........................................................uaucacGgccugcuuugaugagc..............................  28    1  Fm3
........................................................uCucacagccugcuuugaugagc..............................  5     1  Fm3
........................................................uaucacaAccugcuuugaugagc..............................  5     1  Fm3
........................................................uaCcacagccugcuuugaugagc..............................  10    1  Fm3
........................................................uaucacagccugcuuugaCgagc..............................  22    1  Fm3
........................................................uaucacagccugcuuugauAagc..............................  14    1  Fm3
........................................................uaucacagAcugcuuugaugagc..............................  10    1  Fm3
........................................................uaucacagccugcuuugauCagc..............................  3     1  Fm3
........................................................uaucaAagccugcuuugaugagc..............................  4     1  Fm3
........................................................uaucacagccugcuuCgaugagc..............................  7     1  Fm3
........................................................uaucacagccugcuuugaugCgc..............................  3     1  Fm3
........................................................uauGacagccugcuuugaugagc..............................  6     1  Fm3
........................................................uaucacagccugcuuugaugaCc..............................  6     1  Fm3
........................................................uaucacagccGgcuuugaugagc..............................  104   1  Fm3
........................................................uaucaUagccugcuuugaugagc..............................  20    1  Fm3
........................................................uauAacagccugcuuugaugagc..............................  19    1  Fm3
........................................................uaucacagccuAcuuugaugagc..............................  22    1  Fm3
........................................................uaucacagccugcuuuCaugagc..............................  267   1  Fm3
........................................................uaucacagccugcuuugGugagc..............................  245   1  Fm3
........................................................uaucacagcGugcuuugaugagc..............................  3     1  Fm3
........................................................Caucacagccugcuuugaugagc..............................  13    1  Fm3
........................................................uaucacagccCgcuuugaugagc..............................  71    1  Fm3
........................................................Naucacagccugcuuugaugagc..............................  44    1  Fm3
........................................................uaucacagccuUcuuugaugagc..............................  9     1  Fm3
........................................................uaucacagccugcuuugaugagG..............................  2     1  Fm3
........................................................uaucacagccugAuuugaugagc..............................  6     1  Fm3
........................................................uaucacagccugcuuugCugagc..............................  421   1  Fm3
........................................................uaucacagccugUuuugaugagc..............................  120   1  Fm3
........................................................uaucacUgccugcuuugaugagc..............................  4     1  Fm3
........................................................uaucacagccugcuuugGugagcu.............................  2     1  Fm3
........................................................uaucacagccugcuGugaugagcu.............................  1     1  Fm3
........................................................uaucacagccGgcuuugaugagcu.............................  6     1  Fm3
........................................................uaucacUgccugcuuugaugagcu.............................  1     1  Fm3
........................................................uaucacagccugcuuugaugagcC.............................  25    1  Fm3
........................................................uaucacagccugcuuugaugagcG.............................  7     1  Fm3
........................................................uaucacagccugcCuugaugagcu.............................  1     1  Fm3
........................................................uaucacagccugcuuuCaugagcu.............................  4     1  Fm3
........................................................uaucacagccugcuuugaugUgcu.............................  1     1  Fm3
........................................................uaucacagcUugcuuugaugagcu.............................  1     1  Fm3
........................................................uaucacagccugcuuugauAagcu.............................  1     1  Fm3
........................................................uaucacagUcugcuuugaugagcu.............................  2     1  Fm3
........................................................uaucacagccugcuuugaugagUu.............................  7     1  Fm3
........................................................uaGcacagccugcuuugaugagcu.............................  1     1  Fm3
........................................................uaucacNgccugcuuugaugagcu.............................  1     1  Fm3
........................................................uaucacagccugcuuugUugagcu.............................  6     1  Fm3
........................................................uaucacagccugcuuugCugagcu.............................  15    1  Fm3
........................................................uaucCcagccugcuuugaugagcu.............................  3     1  Fm3
........................................................uaucacagccugcuuuAaugagcu.............................  3     1  Fm3
........................................................uaucacagccugcuuugaugagcu.............................  1866  0  Fm3
........................................................uaucGcagccugcuuugaugagcu.............................  1     1  Fm3
........................................................uaucacagccugcuuugauUagcu.............................  2     1  Fm3
........................................................uaucacagccCgcuuugaugagcu.............................  3     1  Fm3
........................................................uaucacagccugcuuugaugagcA.............................  250   1  Fm3
........................................................uaucacagccugcuuuUaugagcu.............................  2     1  Fm3
........................................................Naucacagccugcuuugaugagcu.............................  1     1  Fm3
........................................................uaucacagccugcuuugaugagcuA............................  4     1  Fm3
........................................................uaucacagccugcuuugaugagcuU............................  2     1  Fm3
.........................................................aucacagccugcuuugaugag................................  2     0  Fm3
.........................................................aucacagccugcuuugGugagc...............................  1     1  Fm3
.........................................................aucacagccugcuuugaugagc...............................  13    0  Fm3
..........................................................ucacagccugcuuugaugag................................  3     0  Fm3
..........................................................ucacagccugAuuugaugagc...............................  1     1  Fm3
..........................................................ucacagccugcuuugaugagc...............................  3     0  Fm3
...........................................................cacagccugcuuugaugagc...............................  7     0  Fm3
............................................................acagccugcuuugaugagc...............................  6     0  Fm3

....................ccgucaaagugguugugaug......................................................................  1     0  Mm3
....................ccgucaaaguAguugugaugug....................................................................  1     1  Mm3
```

```
                    Star                                 Mature
uguuugagucuucgucuaguccgucaaagugguugugauguguuauucugacgacauaucacagccugcuuugaugagcuggauguuagaguggacuacagucaaaugc
....................ccgucaaagugguugugaugug....................................................................   18      0    Mm3
....................ccgucaaagugguugugCugug....................................................................   1       1    Mm3
.......................ucaaagugguugugaugug....................................................................   2       0    Mm3
........................................................auaucacagccugcuuugau..................................   1       0    Mm3
........................................................Cuaucacagccugcuuugaugagc..............................   2       1    Mm3
.........................................................uaucacagccugcuuuga...................................   11      0    Mm3
.........................................................uaucacagccugcuuugU...................................   2       1    Mm3
.........................................................uaucacagccugcuuugau..................................   42      0    Mm3
.........................................................uaucacagccugcuuugUu..................................   2       1    Mm3
.........................................................Aaucacagccugcuuugau..................................   1       1    Mm3
.........................................................uaucacagccugcuuugaG..................................   1       1    Mm3
.........................................................uaucacagccugcuuugauA.................................   6       1    Mm3
.........................................................uaucacagUcugcuuugaug.................................   1       1    Mm3
.........................................................uaucacagccugcuuugauU.................................   5       1    Mm3
.........................................................uaucacagccugcuuGgaug.................................   1       1    Mm3
.........................................................uaucacagccugcuuugaug.................................   55      0    Mm3
.........................................................uaucacagccugcuuugaugU................................   1       1    Mm3
.........................................................uaucacagccugcuuugauga................................   49      0    Mm3
.........................................................uaucacagccugcuuugCuga................................   1       1    Mm3
.........................................................uaucacagccuAcuuugauga................................   1       1    Mm3
.........................................................uaucacagUcugcuuugauga................................   2       1    Mm3
.........................................................uaucacagccugUuuugaugag...............................   2       1    Mm3
.........................................................uaucacagccugcuuuCaugag...............................   3       1    Mm3
.........................................................uaucacagccugcuuugUugag...............................   1       1    Mm3
.........................................................uaucacagccugcuuugaugag...............................   370     0    Mm3
.........................................................uaucacagccugcuuugaugUg...............................   1       1    Mm3
.........................................................uaucacagccGgcuuugaugag...............................   1       1    Mm3
.........................................................uaucGcagccugcuuugaugag...............................   1       1    Mm3
.........................................................Naucacagccugcuuugaugag...............................   1       1    Mm3
.........................................................uaNcacagccugcuuugaugag...............................   1       1    Mm3
.........................................................uaucacagccugcuuugaugaU...............................   2       1    Mm3
.........................................................uaucacagccugcCuugaugag...............................   1       1    Mm3
.........................................................uaucacagccugcuuugauUag...............................   2       1    Mm3
.........................................................uaucacagccugcuuugaugaA...............................   1       1    Mm3
.........................................................uaucacagccugcuuugGugag...............................   1       1    Mm3
.........................................................uaucacagccugcuuugCugag...............................   2       1    Mm3
.........................................................uaucacagccCgcuuugaugag...............................   2       1    Mm3
.........................................................uaGcacagccugcuuugaugagc..............................   3       1    Mm3
.........................................................uaCcacagccugcuuugaugagc..............................   4       1    Mm3
.........................................................uauAacagccugcuuugaugagc..............................   4       1    Mm3
.........................................................uaucaNagccugcuuugaugagc..............................   3       1    Mm3
.........................................................uaucacagccCgcuuugaugagc..............................   18      1    Mm3
.........................................................uaucacagccugcCuugaugagc..............................   4       1    Mm3
.........................................................uaucacNgccugcuuugaugagc..............................   1       1    Mm3
.........................................................uaucaUagccugcuuugaugagc..............................   2       1    Mm3
.........................................................uaucacagccugcuuuAaugagc..............................   14      1    Mm3
.........................................................uaucacagccugUuuugaugagc..............................   16      1    Mm3
.........................................................uaucacagccugcuAugaugagc..............................   2       1    Mm3
.........................................................Aaucacagccugcuuugaugagc..............................   1       1    Mm3
.........................................................uaucacagccugcuuugaugaCc..............................   4       1    Mm3
.........................................................uaucacagccugcuuugaugagc..............................   14691   0    Mm3
.........................................................uaAcacagccugcuuugaugagc..............................   5       1    Mm3
.........................................................uaucacagccugcuuGgaugagc..............................   1       1    Mm3
.........................................................uaucacagccugcuuugGugagc..............................   33      1    Mm3
.........................................................uaucacCgccugcuuugaugagc..............................   1       1    Mm3
.........................................................uaucacagccugcuuuCaugagc..............................   39      1    Mm3
.........................................................uaucacagccugcAuugaugagc..............................   6       1    Mm3
.........................................................uaucacagccugAuuugaugagc..............................   3       1    Mm3
.........................................................uaucacagUcugcuuugaugagc..............................   11      1    Mm3
.........................................................uaucacUgccugcuuugaugagc..............................   6       1    Mm3
.........................................................uaucacagccugcuuugaugaAc..............................   6       1    Mm3
.........................................................uaucacagAcugcuuugaugagc..............................   1       1    Mm3
.........................................................uaucacagccugcuuCgaugagc..............................   2       1    Mm3
.........................................................uaucacagcUugcuuugaugagc..............................   4       1    Mm3
.........................................................uCucacagccugcuuugaugagc..............................   1       1    Mm3
.........................................................uaucacagGcugcuuugaugagc..............................   1       1    Mm3
.........................................................uaucacagcAugcuuugaugagc..............................   2       1    Mm3
.........................................................uaucacGgccugcuuugaugagc..............................   16      1    Mm3
.........................................................uaucacagccugcuuugUugagc..............................   58      1    Mm3
.........................................................uaucacaCccugcuuugaugagc..............................   1       1    Mm3
```

```
                    Star                                  Mature
uguuugagucuucgucuaguccgucaaaggugguugugaugugcuuauucugacgacauaucacagccugcuuugaugagcuggauguuagaguggacuacagucaaaugc
..........................................................uaucacagccugcuCugaugagc..............................   1     1   Mm3
..........................................................uaucacaUccugcuuugaugagc..............................   2     1   Mm3
..........................................................uaucacagccugcuuuUaugagc..............................   24    1   Mm3
..........................................................uaucacagccugcuuugaugaUc..............................   7     1   Mm3
..........................................................uaucacagccugcuuugaugagA..............................   13    1   Mm3
..........................................................uaucacagccugcuuugaugagU..............................   65    1   Mm3
..........................................................uaucacagccugcuuugaugUgc..............................   4     1   Mm3
..........................................................uaucacagccugcuuugauUagc..............................   7     1   Mm3
..........................................................uaucacagccGgcuuugaugagc..............................   18    1   Mm3
..........................................................uaucCcagccugcuuugaugagc..............................   2     1   Mm3
..........................................................uaucacagccuUcuuugaugagc..............................   1     1   Mm3
..........................................................uauUacagccugcuuugaugagc..............................   3     1   Mm3
..........................................................Naucacagccugcuuugaugagc..............................   12    1   Mm3
..........................................................uUucacagccugcuuugaugagc..............................   2     1   Mm3
..........................................................uaucacagccugcuuugCugagc..............................   75    1   Mm3
..........................................................uaucacagccugcuuugaCgagc..............................   3     1   Mm3
..........................................................uaucacagccugcuuugauAagc..............................   1     1   Mm3
..........................................................uaucacagccugcuuugaugCgc..............................   1     1   Mm3
..........................................................uauGacagccugcuuugaugagc..............................   5     1   Mm3
..........................................................uaucacagccugcuuAgaugagc..............................   1     1   Mm3
..........................................................Caucacagccugcuuugaugagc..............................   1     1   Mm3
..........................................................uGucacagccugcuuugaugagc..............................   2     1   Mm3
..........................................................uaucacagccCgcuuugaugagcu.............................   2     1   Mm3
..........................................................uaucacagccugcuuugaugCgcu.............................   1     1   Mm3
..........................................................uaucacagccugcuuugaugagcC.............................   3     1   Mm3
..........................................................uaucacagccugUuuugaugagcu.............................   2     1   Mm3
..........................................................uaucacagccugcuuugaCgagcu.............................   3     1   Mm3
..........................................................uaucacagccugcuuugCugagcu.............................   1     1   Mm3
..........................................................uaucacagccugcuuuUaugagcu.............................   1     1   Mm3
..........................................................uaucacagccugcuuugaugagcA.............................   47    1   Mm3
..........................................................uaucacagccugcuuugGugagcu.............................   1     1   Mm3
..........................................................uaucacagccugcuuugaugagcG.............................   6     1   Mm3
..........................................................uaucacagccugcuuuCaugagcu.............................   3     1   Mm3
..........................................................uaAcacagccugcuuugaugagcu.............................   1     1   Mm3
..........................................................uGucacagccugcuuugaugagcu.............................   1     1   Mm3
..........................................................uaucacagUcugcuuugaugagcu.............................   1     1   Mm3
..........................................................uaucacagccugcuuugaugagcu.............................   311   0   Mm3
..........................................................uaucacagccGgcuuugaugagcu.............................   3     1   Mm3
..........................................................uaucacagccugcuuugaugagcuU............................   5     1   Mm3
...........................................................aucacagccugcuuugaugagc..............................   3     0   Mm3
............................................................ucacagccugcuuugaugagcC.............................   1     1   Mm3
..............................................................acagccugcuuugaugagc..............................   2     0   Mm3
...............................................................cagccugcuuugaugagc..............................   1     0   Mm3

.guuugagucuucgucuagu...........................................................................................   5     0   Fm1
....................ccgucaaaggugguugugaugu.....................................................................   4     0   Fm1
....................ccgucaaaggugguugugaugug....................................................................   59    0   Fm1
..........................................................uaucacagccugcuuuga...................................   5     0   Fm1
..........................................................uaucacagUcugcuuugau..................................   2     1   Fm1
..........................................................uaucacagccugcuuugau..................................   54    0   Fm1
..........................................................uaucacagUcugcuuugaug.................................   4     1   Fm1
..........................................................uaucacagccugcuuugauC.................................   1     1   Fm1
..........................................................Caucacagccugcuuugaug.................................   1     1   Fm1
..........................................................uaucacagccugcuuugaug.................................   61    0   Fm1
..........................................................uaucacagccugcuuugauU.................................   7     1   Fm1
..........................................................uaucacagccugcuuugauA.................................   5     1   Fm1
..........................................................uaucacagccugcuuugauga................................   106   0   Fm1
..........................................................uaucacagUcugcuuugauga................................   7     1   Fm1
..........................................................uaucacagccCgcuuugauga................................   1     1   Fm1
..........................................................uaucacagccugcuuugaugU................................   1     1   Fm1
..........................................................uaucacagccugcuuuCaugag...............................   5     1   Fm1
..........................................................uaucacagccugUuuugaugag...............................   13    1   Fm1
..........................................................uaucacagccugcuuuUaugag...............................   2     1   Fm1
..........................................................uaucacagccugcuuugGugag...............................   5     1   Fm1
..........................................................uaucacagccugcuuugaugag...............................   1737  0   Fm1
..........................................................uaucacagccugcuuugUugag...............................   6     1   Fm1
..........................................................uaucacagccugcuuugaCgag...............................   1     1   Fm1
..........................................................uaucacagccugcuuugaugUg...............................   3     1   Fm1
..........................................................uaucacagccugcCuugaugag...............................   1     1   Fm1
..........................................................uaucacagUcugcuuugaugag...............................   9     1   Fm1
```

```
                    Star                                 Mature
uguuugagucuucgucuaguccgucaaagugguugugaugugcuuauucugacgacauaucacagccugcuuugaugagcuggauguuagaguggacuacagucaaaugc
.........................................................uaucacagccugcAuugaugag...............................   1      1   Fm1
.........................................................uaucacagccGgcuuugaugag...............................   7      1   Fm1
.........................................................uaucacagccugcuuugauUag...............................   3      1   Fm1
.........................................................uaucacagccugcuuugCugag...............................   9      1   Fm1
.........................................................uaucacagccugcuuuAaugag...............................   5      1   Fm1
.........................................................uaAcacagccugcuuugaugag...............................   1      1   Fm1
.........................................................uauUacagccugcuuugaugag...............................   1      1   Fm1
.........................................................uaucacUgccugcuuugaugag...............................   1      1   Fm1
.........................................................uaucacagccugcuuugGugagc..............................   224    1   Fm1
.........................................................uaucacagccuCcuuugaugagc..............................   1      1   Fm1
.........................................................uaucacagccugcuuugaugagc..............................   81981  0   Fm1
.........................................................uaucacagccugcuuugaugUgc..............................   10     1   Fm1
.........................................................uCucacagccugcuuugaugagc..............................   2      1   Fm1
.........................................................uGucacagccugcuuugaugagc..............................   22     1   Fm1
.........................................................uaucacagccuAcuuugaugagc..............................   9      1   Fm1
.........................................................uauUacagccugcuuugaugagc..............................   16     1   Fm1
.........................................................uaucCcagccugcuuugaugagc..............................   5      1   Fm1
.........................................................uaucacagccugcuuugauUagc..............................   30     1   Fm1
.........................................................uaucacagccugcuuugauAagc..............................   17     1   Fm1
.........................................................uaucacagccCgcuuugaugagc..............................   75     1   Fm1
.........................................................uaucacagccugcuuuCaugagc..............................   221    1   Fm1
.........................................................uaucacagccugcuuGgaugagc..............................   1      1   Fm1
.........................................................uaucacagccugUuuugaugagc..............................   277    1   Fm1
.........................................................uaucUcagccugcuuugaugagc..............................   6      1   Fm1
.........................................................uaucacGgccugcuuugaugagc..............................   7      1   Fm1
.........................................................uaucaUagccugcuuugaugagc..............................   38     1   Fm1
.........................................................uaucacagccugGuuugaugagc..............................   2      1   Fm1
.........................................................uaucacagccugcuuugaugagU..............................   238    1   Fm1
.........................................................uaucacagUcugcuuugaugagc..............................   168    1   Fm1
.........................................................uaAcacagccugcuuugaugagc..............................   16     1   Fm1
.........................................................uaucacagccugcuuuUaugagc..............................   126    1   Fm1
.........................................................uaucacagAcugcuuugaugagc..............................   4      1   Fm1
.........................................................uaucacagccugcGuugaugagc..............................   4      1   Fm1
.........................................................uaucacagcGugcuuugaugagc..............................   5      1   Fm1
.........................................................Gaucacagccugcuuugaugagc..............................   2      1   Fm1
.........................................................uaucacagccugcuuuAaugagc..............................   147    1   Fm1
.........................................................uaucacagccugcuuugaAgagc..............................   8      1   Fm1
.........................................................uaucacagcAugcuuugaugagc..............................   8      1   Fm1
.........................................................uaucacagccugcuCugaugagc..............................   7      1   Fm1
.........................................................uaucGcagccugcuuugaugagc..............................   15     1   Fm1
.........................................................uaucacagccugcuuugCugagc..............................   383    1   Fm1
.........................................................Caucacagccugcuuugaugagc..............................   17     1   Fm1
.........................................................uaucaNagccugcuuugaugagc..............................   6      1   Fm1
.........................................................uaucacagcUugcuuugaugagc..............................   41     1   Fm1
.........................................................uaucacUgccugcuuugaugagc..............................   13     1   Fm1
.........................................................uaucacaAccugcuuugaugagc..............................   6      1   Fm1
.........................................................Aaucacagccugcuuugaugagc..............................   7      1   Fm1
.........................................................uaucaGagccugcuuugaugagc..............................   4      1   Fm1
.........................................................uaucacagccugcAuugaugagc..............................   9      1   Fm1
.........................................................uaucacagccugcuuugaugagG..............................   4      1   Fm1
.........................................................uaucacagccugcuuugaugaUc..............................   20     1   Fm1
.........................................................uaucacagccugAuuugaugagc..............................   7      1   Fm1
.........................................................uUucacagccugcuuugaugagc..............................   9      1   Fm1
.........................................................uaucacaUccugcuuugaugagc..............................   27     1   Fm1
.........................................................uaucacagccugcuuugaugagA..............................   23     1   Fm1
.........................................................uaucacagGcugcuuugaugagc..............................   2      1   Fm1
.........................................................uaGcacagccugcuuugaugagc..............................   15     1   Fm1
.........................................................uaucacCgccugcuuugaugagc..............................   2      1   Fm1
.........................................................uaucacagccGgcuuugaugagc..............................   109    1   Fm1
.........................................................uauGacagccugcuuugaugagc..............................   10     1   Fm1
.........................................................uNucacagccugcuuugaugagc..............................   1      1   Fm1
.........................................................uaucacagccugcuuAgaugagc..............................   4      1   Fm1
.........................................................uaucacagccugcCuugaugagc..............................   33     1   Fm1
.........................................................uaucaAagccugcuuugaugagc..............................   3      1   Fm1
.........................................................uaucacagccugcuuugauCagc..............................   1      1   Fm1
.........................................................uaucacagccugcuAugaugagc..............................   3      1   Fm1
.........................................................uauAacagccugcuuugaugagc..............................   24     1   Fm1
.........................................................uaucacagccugcuuugaugCgc..............................   1      1   Fm1
.........................................................uaucacagccuUcuuugaugagc..............................   14     1   Fm1
.........................................................uaucacagccugcuGugaugagc..............................   4      1   Fm1
```

Star **Mature**

```
uguuugagucuucgucuaguccgucaaagugguugugaugugcuuauucugacgacauaucacagccugcuuugaugagcuggauguuagaguggacuacagucaaaugc
.........................................................uaucacagccugcuuugUugagc..............................  244   1  Fm1
.........................................................uaucacagccugcuuugaugaCc..............................  5     1  Fm1
.........................................................Naucacagccugcuuugaugagc..............................  52    1  Fm1
.........................................................uaucacagccugcuuugaCgagc..............................  12    1  Fm1
.........................................................uaCcacagccugcuuugaugagc..............................  18    1  Fm1
.........................................................uaucacaCccugcuuugaugagc..............................  2     1  Fm1
.........................................................uaucacagccugcuuugaugaAc..............................  8     1  Fm1
.........................................................uaucacagccugcuuCgaugagc..............................  9     1  Fm1
.........................................................uaucacNgccugcuuugaugagc..............................  4     1  Fm1
.........................................................uaucacagccugcuuugCugagcu.............................  4     1  Fm1
.........................................................uaucacagccugcuuugaugagUu.............................  14    1  Fm1
.........................................................Gaucacagccugcuuugaugagcu.............................  1     1  Fm1
.........................................................uaucacagccugcuuuAaugagcu.............................  1     1  Fm1
.........................................................uaucacagccugcuCugaugagcu.............................  1     1  Fm1
.........................................................uaucacagccGgcuuugaugagcu.............................  5     1  Fm1
.........................................................uaucacagccugcuuuUaugagcu.............................  2     1  Fm1
.........................................................uaucacagUcugcuuugaugagcu.............................  5     1  Fm1
.........................................................uaucacagccugcuuugaCgagcu.............................  3     1  Fm1
.........................................................uaucacagccugcuuugaugUgcu.............................  1     1  Fm1
.........................................................uaucacagccugcuuugaugagcC.............................  28    1  Fm1
.........................................................uaCcacagccugcuuugaugagcu.............................  4     1  Fm1
.........................................................uaucacagccugcuuugaugagcA.............................  77    1  Fm1
.........................................................Naucacagccugcuuugaugagcu.............................  1     1  Fm1
.........................................................uaucacagccugcuuugaugagcG.............................  52    1  Fm1
.........................................................uaucacagccugcuuugGugagcu.............................  2     1  Fm1
.........................................................uaucacaUccugcuuugaugagcu.............................  1     1  Fm1
.........................................................uaucacagccugcuuuCaugagcu.............................  3     1  Fm1
.........................................................uaucacagccugUuuugaugagcu.............................  3     1  Fm1
.........................................................uaucacagccugcuuugaugagcu.............................  1351  0  Fm1
.........................................................uaucacagccCgcuuugaugagcu.............................  4     1  Fm1
.........................................................uaucacagccugcuuugaugagcuU............................  34    1  Fm1
..........................................................aucacagccugcuuugau..................................  2     0  Fm1
..........................................................aucacagccugcuuugaugagc..............................  3     0  Fm1
...........................................................ucacagccugcuuugaugagc..............................  2     0  Fm1
...........................................................ucacagccGgcuuugaugagc..............................  2     1  Fm1
...........................................................ucacagccugcuuugaugagcC.............................  3     1  Fm1
...........................................................ucacagccAgcuuugaugagcug............................  1     1  Fm1

....................ccgucaaagugguugugaug......................................................................  9     0  Fm2
....................ccgucaaagugguugugaugug....................................................................  49    0  Fm2
........................................................auaucacagccugcuuugauga................................  7     0  Fm2
.........................................................uaucacagccugcuuuga...................................  5     0  Fm2
.........................................................uaucacagccugcuuugau..................................  36    0  Fm2
.........................................................uaucacagccugcuuugUu..................................  4     1  Fm2
.........................................................uaucacagccugcuuugUug.................................  1     1  Fm2
.........................................................uaucacagccugUuuugaug.................................  8     1  Fm2
.........................................................uaucacagccugcuuugauU.................................  2     1  Fm2
.........................................................uaucacagccugcuuGgaug.................................  1     1  Fm2
.........................................................uaucacagccugcuuugaug.................................  59    0  Fm2
.........................................................uaucacagccugcuuugCuga................................  2     1  Fm2
.........................................................uaucacagccugcuuugUuga................................  1     1  Fm2
.........................................................uaucacagccugcuuugauga................................  95    0  Fm2
.........................................................uaGcacagccugcuuugauga................................  1     1  Fm2
.........................................................uaucacagccugcuuugGuga................................  1     1  Fm2
.........................................................uaucacagccugcuuugCugag...............................  2     1  Fm2
.........................................................uaucacagccCgcuuugaugag...............................  1     1  Fm2
.........................................................uaucacagccugcuuuUaugag...............................  3     1  Fm2
.........................................................uaucacagccugcuuugaugaU...............................  1     1  Fm2
.........................................................uaucacagccugcuuugaugUg...............................  4     1  Fm2
.........................................................uaucacagccugGuuugaugag...............................  1     1  Fm2
.........................................................uaucacagccugcuCugaugag...............................  1     1  Fm2
.........................................................uaucacagccugcuuugGugag...............................  1     1  Fm2
.........................................................uaucacGgccugcuuugaugag...............................  1     1  Fm2
.........................................................uaucacagccugcuuugauUag...............................  6     1  Fm2
.........................................................uaucacagccugcuuuCaugag...............................  2     1  Fm2
.........................................................uaucacagccugcuuugaugCg...............................  1     1  Fm2
.........................................................uaucacagccugcuuugaugag...............................  545   0  Fm2
.........................................................uaucacagccugcuuugauAag...............................  1     1  Fm2
.........................................................uaucacagccugcuuugUugag...............................  2     1  Fm2
.........................................................Naucacagccugcuuugaugag...............................  1     1  Fm2
```

```
                    Star                                      Mature
uguuugagucuucgucuaguccgucaaagugguugugaugugcuuauucugacgacauaucacagccugcuuugaugagcuggauguuagaguggacuacagucaaaugc
.........................................................uaucacagcUugcuuugaugagc..............................    6        1    Fm2
.........................................................uaucacagccugcuuugauAagc..............................    12       1    Fm2
.........................................................uaucacagccugcuuugaGgagc..............................    1        1    Fm2
.........................................................uCucacagccugcuuugaugagc..............................    6        1    Fm2
.........................................................uaucaNagccugcuuugaugagc..............................    19       1    Fm2
.........................................................uaucacagccugcuuugaugagU..............................    65       1    Fm2
.........................................................uaucacaCccugcuuugaugagc..............................    3        1    Fm2
.........................................................uaucacagccuAcuuugaugagc..............................    8        1    Fm2
.........................................................uaucacagccugcuuugaugaCc..............................    2        1    Fm2
.........................................................uaucacagccugcAuugaugagc..............................    17       1    Fm2
.........................................................uaucacagccugcuuugCugagc..............................    333      1    Fm2
.........................................................uaucacagccugcuAugaugagc..............................    7        1    Fm2
.........................................................uaucacagcGugcuuugaugagc..............................    6        1    Fm2
.........................................................uaucacagccugcuuugaCgagc..............................    17       1    Fm2
.........................................................uaucacagccugcuuugaugUgc..............................    10       1    Fm2
.........................................................uaucacagAcugcuuugaugagc..............................    3        1    Fm2
.........................................................uaucacagccCgcuuugaugagc..............................    79       1    Fm2
.........................................................uaucCcagccugcuuugaugagc..............................    11       1    Fm2
.........................................................uaucacagccugcuuugaugagG..............................    3        1    Fm2
.........................................................uaucacUgccugcuuugaugagc..............................    23       1    Fm2
.........................................................uaucacagccuUcuuugaugagc..............................    8        1    Fm2
.........................................................uaucacagccugGuuugaugagc..............................    1        1    Fm2
.........................................................uaucacagccugcuCugaugagc..............................    5        1    Fm2
.........................................................uaucacGgccugcuuugaugagc..............................    49       1    Fm2
.........................................................uaucacagccugcuuCgaugagc..............................    10       1    Fm2
.........................................................uaucacagGcugcuuugaugagc..............................    3        1    Fm2
.........................................................uaucacagccugcuuugauCagc..............................    10       1    Fm2
.........................................................uaucacagccugUuuugaugagc..............................    201      1    Fm2
.........................................................uaucacagccugcuuAgaugagc..............................    5        1    Fm2
.........................................................uaucacagccugAuuugaugagc..............................    3        1    Fm2
.........................................................uaucacagccugcuuugUugagc..............................    290      1    Fm2
.........................................................uauGacagccugcuuugaugagc..............................    14       1    Fm2
.........................................................uaucacagccugcuuugaugagc..............................    85131    0    Fm2
.........................................................uaucacagccugcuuugaAgagc..............................    6        1    Fm2
.........................................................uauAacagccugcuuugaugagc..............................    30       1    Fm2
.........................................................uaucacagccugcuuuAaugagc..............................    175      1    Fm2
.........................................................uaucacagccugcuGugaugagc..............................    6        1    Fm2
.........................................................uaucacagccugcuuugaugaUc..............................    14       1    Fm2
.........................................................uaucacagccugcuuugaugaAc..............................    9        1    Fm2
.........................................................uaucacagccugcCuugaugagc..............................    59       1    Fm2
.........................................................uaGcacagccugcuuugaugagc..............................    22       1    Fm2
.........................................................uaucacagccugcuuugauUagc..............................    28       1    Fm2
.........................................................uaucacagccugcuuuCaugagc..............................    235      1    Fm2
.........................................................uauUacagccugcuuugaugagc..............................    22       1    Fm2
.........................................................uaucacagUcugcuuugaugagc..............................    101      1    Fm2
.........................................................uNucacagccugcuuugaugagc..............................    1        1    Fm2
.........................................................uaucacagccugcuuugaugagA..............................    4        1    Fm2
.........................................................uaucacaAccugcuuugaugagc..............................    10       1    Fm2
.........................................................uaucacagccGgcuuugaugagc..............................    101      1    Fm2
.........................................................uUucacagccugcuuugaugagc..............................    16       1    Fm2
.........................................................uaucGcagccugcuuugaugagc..............................    16       1    Fm2
.........................................................uaucacagccugcuuuUaugagc..............................    129      1    Fm2
.........................................................uaucaGagccugcuuugaugagc..............................    2        1    Fm2
.........................................................Caucacagccugcuuugaugagc..............................    17       1    Fm2
.........................................................uaucacagccugcGuugaugagc..............................    8        1    Fm2
.........................................................uaucacagccugcuuugaugCgc..............................    2        1    Fm2
.........................................................uaucacagccugcuuGgaugagc..............................    5        1    Fm2
.........................................................uaucacNgccugcuuugaugagc..............................    7        1    Fm2
.........................................................uaucUcagccugcuuugaugagc..............................    12       1    Fm2
.........................................................uaCcacagccugcuuugaugagc..............................    13       1    Fm2
.........................................................uaucacaUccugcuuugaugagc..............................    24       1    Fm2
.........................................................uaucacCgccugcuuugaugagc..............................    5        1    Fm2
.........................................................uaucaUagccugcuuugaugagc..............................    23       1    Fm2
.........................................................Naucacagccugcuuugaugagc..............................    39       1    Fm2
.........................................................uaucacagccugcuuugGugagc..............................    212      1    Fm2
.........................................................uaAcacagccugcuuugaugagc..............................    20       1    Fm2
.........................................................uaucacagcAugcuuugaugagc..............................    5        1    Fm2
.........................................................uGucacagccugcuuugaugagc..............................    12       1    Fm2
.........................................................Aaucacagccugcuuugaugagc..............................    2        1    Fm2
.........................................................uaucacagccugcuuuUaugagcu.............................    3        1    Fm2
```

Star **Mature**

```
uguuugagucuucgucuagu ccgucaaagugguugugaugug cuuauucugacgaca uaucacagccugcuuugaugagc uggauguuagaguggacuacagucaaaugc
.........................................................uaucacagccugcuuugaugaUcu.............................   1     1  Fm2
.........................................................uaucacagccugcuuugCugagcu.............................   4     1  Fm2
.........................................................uaucacagccugcuuugaugaCcu.............................   1     1  Fm2
.........................................................uaucacagccugcuuugaugagcG.............................   57    1  Fm2
.........................................................uauAacagccugcuuugaugagcu.............................   1     1  Fm2
.........................................................uaucacagccugUuuugaugagcu.............................   4     1  Fm2
.........................................................Naucacagccugcuuugaugagcu.............................   1     1  Fm2
.........................................................uaucacagccugcuuugGugagcu.............................   4     1  Fm2
.........................................................uaucacagccugcuuugaugagcC.............................   68    1  Fm2
.........................................................uaucacagccugcuuugaugagcA.............................   111   1  Fm2
.........................................................uaucacagccGgcuuugaugagcu.............................   10    1  Fm2
.........................................................uaucacagccugcuuuCaugagcu.............................   3     1  Fm2
.........................................................uaucacagccugcuuugaugagcu.............................   1269  0  Fm2
.........................................................uaucacagccugcuuugUugagcu.............................   4     1  Fm2
.........................................................uaucacagccugcuuuAaugagcu.............................   4     1  Fm2
..........................................................Uucacagccugcuuugaugagcu.............................   1     1  Fm2
...........................................................ucacagccGgcuuugaugagc..............................   7     1  Fm2
...........................................................ucacagccugcuuugaugagcC.............................   2     1  Fm2

.guuugagucuucgucuagu..........................................................................................   1     0  Mm2
....................ccgucaaagugguugugaugug....................................................................   40    0  Mm2
....................ccgucaaagugguugugaugugA...................................................................   1     1  Mm2
..........................................cuuauucugacgacauaucacagccugcuuugaugagc..............................   3     0  Mm2
.........................................................uaucacagccugcuuugU...................................   2     1  Mm2
.........................................................uaucacagccugcuuuga...................................   22    0  Mm2
.........................................................uaucacagccugcuuuUau..................................   1     1  Mm2
.........................................................uaucacagccugcuuugaA..................................   2     1  Mm2
.........................................................uaucacagccugcuuuCau..................................   1     1  Mm2
.........................................................uaucacagccugcuuugUu..................................   6     1  Mm2
.........................................................uaucacagccugcuuugau..................................   35    0  Mm2
.........................................................uaucacagccugcuuuAau..................................   3     1  Mm2
.........................................................uaucacaUccugcuuugau..................................   1     1  Mm2
.........................................................uaucacagccugcuuugauA.................................   3     1  Mm2
.........................................................uaucacagccugcuuuUaug.................................   1     1  Mm2
.........................................................uaucacagccugcuuugaug.................................   65    0  Mm2
.........................................................uaucacagccugcuuugauU.................................   1     1  Mm2
.........................................................uaucacagccugcuuugGuga................................   1     1  Mm2
.........................................................uaucacagccugcuuugaugU................................   2     1  Mm2
.........................................................uaucacagccugcuuugauga................................   97    0  Mm2
.........................................................uaucacagccugcuuugaugC................................   2     1  Mm2
.........................................................uaucacagUcugcuuugauga................................   2     1  Mm2
.........................................................uaucacagccugAuuugaugag...............................   1     1  Mm2
.........................................................uaucacagUcugcuuugaugag...............................   8     1  Mm2
.........................................................uaucacGgccugcuuugaugag...............................   2     1  Mm2
.........................................................uaucacagccugcuuugCugag...............................   3     1  Mm2
.........................................................uaucacagccugcuuuCaugag...............................   6     1  Mm2
.........................................................uaucacagccugcuuugauAag...............................   1     1  Mm2
.........................................................uaucacagccugcuuugUugag...............................   5     1  Mm2
.........................................................uaucacagccugUuuugaugag...............................   5     1  Mm2
.........................................................uaucacagccugcuuugaugUg...............................   1     1  Mm2
.........................................................uaucacagccugcuuugaugaA...............................   1     1  Mm2
.........................................................uaucacagccGgcuuugaugag...............................   1     1  Mm2
.........................................................uaucacagccugcuuugGugag...............................   4     1  Mm2
.........................................................uaucacagccugcuuugauUag...............................   3     1  Mm2
.........................................................uaucacagccugcuuugaugag...............................   1196  0  Mm2
.........................................................uaucacagccugcuuGgaugag...............................   1     1  Mm2
.........................................................uaucacagccugcuuuAaugag...............................   2     1  Mm2
.........................................................uaucaUagccugcuuugaugag...............................   1     1  Mm2
.........................................................uaucacagccCgcuuugaugag...............................   1     1  Mm2
.........................................................uaucacagccugcuuugGugagc..............................   246   1  Mm2
.........................................................uaucacagccugcuuGgaugagc..............................   5     1  Mm2
.........................................................uaucacagccCgcuuugaugagc..............................   56    1  Mm2
.........................................................Aaucacagccugcuuugaugagc..............................   6     1  Mm2
.........................................................uaCcacagccugcuuugaugagc..............................   9     1  Mm2
.........................................................uaucacagccugcuuugaugagA..............................   42    1  Mm2
.........................................................uaucacagccugcuuuAaugagc..............................   167   1  Mm2
.........................................................uaucacagccugcuCugaugagc..............................   8     1  Mm2
.........................................................uauAacagccugcuuugaugagc..............................   33    1  Mm2
.........................................................uauGacagccugcuuugaugagc..............................   6     1  Mm2
.........................................................uUucacagccugcuuugaugagc..............................   11    1  Mm2
```

```
                    Star                                    Mature
uguuugagucuucgucuaguccgucaaagugguugugaugugcuuauucugacgacauaucacagccugcuuugaugagcuggauguuagaguggacuacagucaaaugc
.........................................................uaucacagccugcuuugaGgagc..............................   3      1   Mm2
.........................................................uaucGcagccugcuuugaugagc..............................   16     1   Mm2
.........................................................uaucacagccugcuuuUaugagc..............................   139    1   Mm2
.........................................................uaAcacagccugcuuugaugagc..............................   15     1   Mm2
.........................................................uaucacaAccugcuuugaugagc..............................   7      1   Mm2
.........................................................uaucaUagccugcuuugaugagc..............................   22     1   Mm2
.........................................................uaucaNagccugcuuugaugagc..............................   8      1   Mm2
.........................................................uGucacagccugcuuugaugagc..............................   8      1   Mm2
.........................................................Caucacagccugcuuugaugagc..............................   10     1   Mm2
.........................................................uaucacagcGugcuuugaugagc..............................   2      1   Mm2
.........................................................Gaucacagccugcuuugaugagc..............................   1      1   Mm2
.........................................................uaucacagccuAcuuugaugagc..............................   9      1   Mm2
.........................................................uaucacagUcugcuuugaugagc..............................   45     1   Mm2
.........................................................uaucacagccugcAuugaugagc..............................   12     1   Mm2
.........................................................uaucCcagccugcuuugaugagc..............................   11     1   Mm2
.........................................................uaucacagccuUcuuugaugagc..............................   5      1   Mm2
.........................................................uaucacGgccugcuuugaugagc..............................   19     1   Mm2
.........................................................uaucacagccugcuuugaugagG..............................   6      1   Mm2
.........................................................uaucacagccugcuuAgaugagc..............................   3      1   Mm2
.........................................................uaucacagccugcuuugCugagc..............................   359    1   Mm2
.........................................................uaucacagccugcuuugaugagc..............................   81637  0   Mm2
.........................................................uaucacagccugcuuugaCgagc..............................   14     1   Mm2
.........................................................uaucacUgccugcuuugaugagc..............................   13     1   Mm2
.........................................................uaucacagccugUuuugaugagc..............................   70     1   Mm2
.........................................................uaucacagAcugcuuugaugagc..............................   3      1   Mm2
.........................................................uaucacagccGgcuuugaugagc..............................   130    1   Mm2
.........................................................uaucacagccugcuuugaugUgc..............................   16     1   Mm2
.........................................................uaucacagccugcuuugaAgagc..............................   9      1   Mm2
.........................................................uaucacagccugcuuuCaugagc..............................   231    1   Mm2
.........................................................uaucacagccugcuuugaugaAc..............................   10     1   Mm2
.........................................................uaucacagGcugcuuugaugagc..............................   3      1   Mm2
.........................................................uaucacaCccugcuuugaugagc..............................   4      1   Mm2
.........................................................uaucacagccugcuuugaugagU..............................   152    1   Mm2
.........................................................uaucacagcAugcuuugaugagc..............................   5      1   Mm2
.........................................................uaucacagccugcuAugaugagc..............................   4      1   Mm2
.........................................................uaucacCgccugcuuugaugagc..............................   3      1   Mm2
.........................................................uaucacagccugcGuugaugagc..............................   10     1   Mm2
.........................................................uaucacagccugcuuugaugCgc..............................   5      1   Mm2
.........................................................uaucacagccugcuuugaugaCc..............................   3      1   Mm2
.........................................................uaucacagccugcuuugUugagc..............................   264    1   Mm2
.........................................................uaucUcagccugcuuugaugagc..............................   14     1   Mm2
.........................................................uaucacagccugcuuugauAagc..............................   10     1   Mm2
.........................................................uaucacagccugAuuugaugagc..............................   4      1   Mm2
.........................................................uaucacagccugcCuugaugagc..............................   30     1   Mm2
.........................................................Naucacagccugcuuugaugagc..............................   38     1   Mm2
.........................................................uaucacagccuCcuuugaugagc..............................   1      1   Mm2
.........................................................uaucacagccugcuGugaugagc..............................   1      1   Mm2
.........................................................uCucacagccugcuuugaugagc..............................   9      1   Mm2
.........................................................uaucaGagccugcuuugaugagc..............................   2      1   Mm2
.........................................................uaucacagccugcuuugaugaUc..............................   15     1   Mm2
.........................................................uaGcacagccugcuuugaugagc..............................   25     1   Mm2
.........................................................uauUacagccugcuuugaugagc..............................   13     1   Mm2
.........................................................uaucacagccugcuuugauCagc..............................   9      1   Mm2
.........................................................uaucacagccugGuuugaugagc..............................   2      1   Mm2
.........................................................uaucacNgccugcuuugaugagc..............................   1      1   Mm2
.........................................................uaucacagccugcuuugauUagc..............................   20     1   Mm2
.........................................................uaucacagcUugcuuugaugagc..............................   14     1   Mm2
.........................................................uaucacaUccugcuuugaugagc..............................   27     1   Mm2
.........................................................uaucacagccugcuuCgaugagc..............................   7      1   Mm2
.........................................................uaNcacagccugcuuugaugagc..............................   1      1   Mm2
.........................................................uaucacagUcugcuuugaugagcu.............................   1      1   Mm2
.........................................................uaucacagccugcuuuUaugagcu.............................   1      1   Mm2
.........................................................uaucacagccugcuuugaugagUu.............................   7      1   Mm2
.........................................................uaucacagccugcuuuCaugagcu.............................   4      1   Mm2
.........................................................uaucacagccugcuuugaugagcC.............................   25     1   Mm2
.........................................................uaucacagccugcuuugaugagcA.............................   152    1   Mm2
.........................................................uaucacagccugcuuugaugagGu.............................   1      1   Mm2
.........................................................uaucacagccCgcuuugaugagcu.............................   3      1   Mm2
.........................................................uaucacagccGgcuuugaugagcu.............................   5      1   Mm2
.........................................................uaucacagccugcuuugCugagcu.............................   6      1   Mm2
```

```
                    Star                                   Mature
uguuugagucuucgucuaguccgucaaagugguugugaugugcuuauucugacgacauaucacagccugcuuugaugagcuggauguuagaguggacuacagucaaaugc
.........................................................uaucacagccugcuuugaugagcu.............................    1220   0   Mm2
.........................................................uaucacagccugcuuugGugagcu.............................    1      1   Mm2
.........................................................uaucGcagccugcuuugaugagcu.............................    1      1   Mm2
.........................................................uaucacagccugcAuugaugagcu.............................    2      1   Mm2
.........................................................uaucacagccuAcuuugaugagcu.............................    2      1   Mm2
.........................................................uaucacagccugcuuugaugagcG.............................    33     1   Mm2
.........................................................uaucacagccugcuuuAaugagcu.............................    4      1   Mm2
.........................................................uaucacagccugcuuugaCgagcu.............................    3      1   Mm2
.........................................................Caucacagccugcuuugaugagcu.............................    1      1   Mm2
.........................................................uaucacagccugcuuugUugagcu.............................    2      1   Mm2
.........................................................uaucacagccugcuuugaugagcug............................    1      0   Mm2
.........................................................uaucacagccugcuuugaugagcuU............................    22     1   Mm2
.........................................................uaucacagccugcuuugaugagcuA............................    2      1   Mm2
..........................................................aucacagccugcuuugaugagc..............................    13     0   Mm2
..........................................................aucacagccugcuuugaugUgc..............................    1      1   Mm2
...........................................................ucacagccGgcuuugaugagc..............................    3      1   Mm2
...........................................................ucacagccugcuuugaugagc..............................    1      0   Mm2
...........................................................ucacagccugcuuugaugagcC.............................    1      1   Mm2
..............................................................cagccugcuuugaugagc..............................    1      0   Mm2

....................ccgucaaagugguugugaugug....................................................................    9      0   Mm1
....................ccgucaaagugguugugUugug....................................................................    1      1   Mm1
........................................................Cuaucacagccugcuuugaugagc..............................    3      1   Mm1
.........................................................uaucacagccugcuuuCa...................................    1      1   Mm1
.........................................................uaucacagccugcuuugU...................................    1      1   Mm1
.........................................................uaucacagccugcuuuga...................................    36     0   Mm1
.........................................................uaucacagccugcuuAga...................................    1      1   Mm1
.........................................................uaucacagccugcuuugUu..................................    7      1   Mm1
.........................................................uaucacagccugcuuugaug.................................    16     0   Mm1
.........................................................uaucacagccugcuuGgaug.................................    1      1   Mm1
.........................................................uaucacagccugcuuugauga................................    11     0   Mm1
.........................................................uaucacagccugcuuugaugU................................    4      1   Mm1
.........................................................uaucacGgccugcuuugaugag...............................    13     1   Mm1
.........................................................uaucacagccugcuuugGugag...............................    1      1   Mm1
.........................................................uaucacagccugcuuugauUag...............................    1      1   Mm1
.........................................................uaucacagccGgcuuugaugag...............................    1      1   Mm1
.........................................................uaucacagccugcuuuCaugag...............................    2      1   Mm1
.........................................................uaucacagccugcuuGgaugag...............................    1      1   Mm1
.........................................................uaucacagccugcuuugaugag...............................    186    0   Mm1
.........................................................uaucacagccugcuuugUugag...............................    1      1   Mm1
.........................................................uaucacagccugcuuCgaugagc..............................    2      1   Mm1
.........................................................uauUacagccugcuuugaugagc..............................    2      1   Mm1
.........................................................uGucacagccugcuuugaugagc..............................    2      1   Mm1
.........................................................uaucaNagccugcuuugaugagc..............................    1      1   Mm1
.........................................................Caucacagccugcuuugaugagc..............................    1      1   Mm1
.........................................................uaucacagccugcuuugaugaUc..............................    2      1   Mm1
.........................................................uaGcacagccugcuuugaugagc..............................    1      1   Mm1
.........................................................uaucacUgccugcuuugaugagc..............................    3      1   Mm1
.........................................................uaucacagccugcuGugaugagc..............................    1      1   Mm1
.........................................................uaucacagccugcuuugUugagc..............................    39     1   Mm1
.........................................................uaucacagccugcAuugaugagc..............................    3      1   Mm1
.........................................................uaucacagccugcuuuUaugagc..............................    20     1   Mm1
.........................................................uUucacagccugcuuugaugagc..............................    2      1   Mm1
.........................................................uaucacagccuUcuuugaugagc..............................    2      1   Mm1
.........................................................uaucCcagccugcuuugaugagc..............................    1      1   Mm1
.........................................................uaucacagccugcuuugGugagc..............................    34     1   Mm1
.........................................................uaucacagccugcuuugaAgagc..............................    1      1   Mm1
.........................................................uaucacagccuAcuuugaugagc..............................    2      1   Mm1
.........................................................uaucacagccugUuuugaugagc..............................    14     1   Mm1
.........................................................uaucacagccugcGuugaugagc..............................    1      1   Mm1
.........................................................uaucacagccugcuuugauAagc..............................    5      1   Mm1
.........................................................uaAcacagccugcuuugaugagc..............................    3      1   Mm1
.........................................................uaucacagccugcuuugaugagc..............................    12931  0   Mm1
.........................................................uaCcacagccugcuuugaugagc..............................    2      1   Mm1
.........................................................uaucacagccugcuAugaugagc..............................    1      1   Mm1
.........................................................uaucacagcAugcuuugaugagc..............................    1      1   Mm1
.........................................................uaucacNgccugcuuugaugagc..............................    2      1   Mm1
.........................................................uaucacagUcugcuuugaugagc..............................    31     1   Mm1
.........................................................uaucacaUccugcuuugaugagc..............................    5      1   Mm1
.........................................................uaucacagccGgcuuugaugagc..............................    12     1   Mm1
```

```
                    Star                                 Mature
uguuugagucuucgucuaguccgucaaagugguugugaugugcuuauucugacgacauaucacagccugcuuugaugagcuggauguuagaguggacuacagucaaaugc
.........................................................uaucGcagccugcuuugaugagc..............................   2    1   Mm1
.........................................................uaucacagccugcCuugaugagc..............................   2    1   Mm1
.........................................................uaucacGgccugcuuugaugagc..............................   148  1   Mm1
.........................................................uaucaUagccugcuuugaugagc..............................   3    1   Mm1
.........................................................uaucacagccugcuuuCaugagc..............................   28   1   Mm1
.........................................................uaucacCgccugcuuugaugagc..............................   1    1   Mm1
.........................................................uaucacagccugcuuugauUagc..............................   4    1   Mm1
.........................................................uaucacagccugcuuuAaugagc..............................   15   1   Mm1
.........................................................uaucUcagccugcuuugaugagc..............................   1    1   Mm1
.........................................................uauAacagccugcuuugaugagc..............................   3    1   Mm1
.........................................................Naucacagccugcuuugaugagc..............................   7    1   Mm1
.........................................................uaucacagccugcuuugaugagU..............................   52   1   Mm1
.........................................................uaucacagccugcuCugaugagc..............................   1    1   Mm1
.........................................................uCucacagccugcuuugaugagc..............................   2    1   Mm1
.........................................................uaucacagccCgcuuugaugagc..............................   6    1   Mm1
.........................................................Gaucacagccugcuuugaugagc..............................   2    1   Mm1
.........................................................uaucacagccugcuuugaCgagc..............................   5    1   Mm1
.........................................................uaucacagcUugcuuugaugagc..............................   2    1   Mm1
.........................................................uaucacagccugcuuugaugUgc..............................   5    1   Mm1
.........................................................uaucacagcGugcuuugaugagc..............................   2    1   Mm1
.........................................................uaucacagccugcuuugCugagc..............................   65   1   Mm1
.........................................................uaucaNagccugcuuugaugagcu.............................   1    1   Mm1
.........................................................uaucacagccugcuuugaugagcu.............................   208  0   Mm1
.........................................................uaucacagccugcuuuUaugagcu.............................   1    1   Mm1
.........................................................uaucacagccugcuuugaugagcA.............................   16   1   Mm1
.........................................................uaucacagccugcuuugUugagcu.............................   1    1   Mm1
.........................................................uaucacGgccugcuuugaugagcu.............................   18   1   Mm1
.........................................................uaucacagccCgcuuugaugagcu.............................   3    1   Mm1
.........................................................uGucacagccugcuuugaugagcu.............................   1    1   Mm1
.........................................................uaucacagccugcuuugaugagcG.............................   1    1   Mm1
.........................................................uaucacagccGgcuuugaugagcu.............................   4    1   Mm1
.........................................................uaucacagccugcuuugaugagcC.............................   7    1   Mm1
.........................................................uaucacagccugcuuugaugagcuC............................   9    1   Mm1
..........................................................aucacagccugcuuugaugagc..............................   8    0   Mm1
```
